# The Impact of Adding Sunflower Seed Oil Bodies to a Sugar-Free Plant-Based Ice Cream Formulation

**DOI:** 10.3390/foods15030472

**Published:** 2026-01-29

**Authors:** Flavius George Viorel, Cristian Szekely, Andruța Elena Mureșan, Andreea Pușcaș, Vlad Mureșan

**Affiliations:** Food Engineering Department, Faculty of Food Science and Technology, University of Agricultural Sciences and Veterinary Medicine Cluj-Napoca, 400394 Cluj-Napoca, Romania

**Keywords:** plant-based ice cream, oilseeds, oleosomes, natural emulsifiers

## Abstract

The increasing demand for plant-based alternatives, driven by veganism, lactose intolerance, and greater health consciousness, has intensified research into dairy-free frozen desserts. This study investigates the development of a plant-based ice cream alternative utilizing oleosomes extracted from sunflower seed kernels as natural emulsifiers, eliminating the need for synthetic additives. Oleosomes were obtained through aqueous extraction from raw kernels, incorporated into emulsions in three levels (0, 12, and 24%), and combined with sunflower seed oil, tahini, date paste, and water to create the ice cream (IC) formulations. The physicochemical properties of three formulations of a sugar-free frozen dessert were studied. Physicochemical analyses assessed nutritional value, color (CIELab), melting time, stability, overrun, viscosity, and texture profile (TPA). Sensory evaluation was conducted using a hedonic test to assess the impact of tahini type (sunflower seed tahini or pumpkin seed kernel tahini) on the product acceptance. Results showed that higher oleosome content improved emulsion stability and melting resistance, while also producing a softer (30.74 ± 0.28 N), less adhesive (1.87 ± 0.20 mJ) texture, suitable for plant-based ice cream. Sensory analysis revealed a clear preference for the pumpkin tahini formulation, which scored 8.21 ± 0.62 for overall appreciation. The findings demonstrate that the addition of oleosome might improve textural attributes of the products, while the consumer preference could also be influenced by the type of tahini involved in the formulation. However, further studies are necessary to corroborate the proposed interaction mechanisms of ingredients.

## 1. Introduction

The emergence of vegetarianism and veganism in recent years has changed consumer choices in the global food market. Increased awareness of health, sustainability, and ethical considerations related to animal welfare are the major factors driving this change, according to Lea, Crawford [1]. Medical conditions, including lactose intolerance, milk protein allergies, or other dietary restrictions that limit the use of traditional dairy products, affect many people. It is estimated that over 57–65% of adults worldwide suffer from lactose intolerance, highlighting the need for plant-based alternatives [2].

Traditional ice cream (IC) ingredients typically include milk, cream, and sugar, as well as stabilizers and emulsifiers [3]. Although these additives are useful for obtaining a stable product, they are often less attractive to consumers, who are turning their attention to clean-label products [4].

Plant-based IC differs substantially from other plant-based dairy alternatives (e.g., beverages, yogurts, and cheeses) because of its complicated physicochemical structure and processing requirements. Unlike beverages (simple emulsions) or yogurts (protein gels), IC is a complex colloidal system consisting of ice crystals, air bubbles, and partially coalesced fat globules within a concentrated unfrozen matrix [5,6]. Frozen desserts must achieve uniform air incorporation (overrun), resistance to melting, and structural integrity during frozen storage, in addition to being nutritious and palatable [3].

Sunflower (*Helianthus annuus* L.) is a valuable oilseed plant, widely cultivated for its seeds that are rich in unsaturated fats, proteins, fiber, vitamins, and minerals, and is also a promising raw material for plant-based foods [7,8,9]. The high protein content, mainly albumins and globulins, promotes emulsification and aeration, and bioactive compounds such as vitamin E and phytosterols contribute to the oxidative stability of the products [8,9]. The delicate aroma and light color of the seeds allow for easy integration into products such as frozen desserts, without negatively affecting the sensory profile [9]. Romania occupies an important place in global sunflower production, ranking 4th globally in 2018–2023, with an annual average of 2.6 million tons [10], which supports the local availability of raw materials.

Native lipid structures called oleosomes are present in fat-rich seeds such as sunflower seeds. They have intrinsic emulsifying qualities and physical stability due to their triglyceride core, which is surrounded by a membrane of phospholipids and proteins [7]. Oleosomes are attractive components for the development of innovative products due to their structure, which offers them emulsifying capacity [11].

The applicability of oleosomes in the food industry is increasingly studied, due to their usage in obtaining alternatives to dairy products such as IC, cheese, yogurt, and milk, contributing to better stability, texture, and nutritional value [12]. Despite their recognized advantages, industrial applicability is limited by the low yields of conventional extraction (55–60%) and the high costs of enzymes or high-energy technologies. Commercial viability depends on the optimization of scalable methods, such as twin-screw pressing or hybrid systems, capable of maintaining the structural integrity of oleosomes under industrial conditions. Proper scaling can significantly reduce energy losses compared to extraction on a laboratory scale, although the main problems include the difficulty of maintaining the structural integrity of the phospholipid–protein monolayer under mechanical stress, the high cost of enzyme preparations, and the expense of specialized equipment [13].

The use of oleosomes in systems involving freezing, such as IC, presents major challenges because repeated freeze–thaw cycles tend to destabilize these emulsions, a process that can lead to oil release and loss of structural integrity [14]. At −20 °C, partial coalescence is the primary cause of oleosome rupture [15]. This vulnerability is critical to product quality, as it is accompanied by variability in the size and composition of oleosomes, depending on the botanical source and the extraction method, factors that directly affect their physicochemical stability and mechanical properties. In addition, their compatibility with other commercial ingredients in complex food matrices remain unresolved technological challenges that requires further research [14].

In the current paper, the properties of an innovative frozen dessert formulated based on sunflower seed oleosomes, vegetable oil, tahini, and date paste are described. The main goal was to use oleosomes extracted from oilseeds as structuring agents in sugar-free, vegan frozen desserts to replace both fat and synthetic emulsifiers and to obtain a nutritionally improved plant-based product. By imitating milk fat globules, we hypothesized that adding more oleosomes would improve the textural and rheological properties of the samples. Date paste was included due to its high carbohydrate and fiber content, as a sweetening component [16]. Two different variants of tahini were used, aiming to determine the IC level of consumer acceptability in relation to the tahini used.

While oil bodies have been explored as fat replacers in IC, for example, using peanut oil bodies demonstrates the potential of oleosomes (fresh, heated, or roasted) as a fat substitute in plant-based IC, the application of native sunflower oleosomes in plant-based IC remains underexplored, especially in vegan and sugar-free frozen desserts. This study addresses this gap, by evaluating the contribution of sunflower oleosomes to the physicochemical and textural properties of plant-based IC formulated without animal-derived products, added sugar, or emulsifiers [17].

The resulting product is intended for vegan and vegetarian consumers, as well as for people with lactose intolerance or those who follow a dairy-free diet for health or personal reasons.

## 2. Materials and Methods

### 2.1. Tahini

To obtain the oleaginous seed paste (tahini), 1500 g of raw sunflower seed kernels (Best Nuts SRL, Timiș, Romania) or raw pumpkin seed kernels (Solaris Plant SRL, Bucharest, Romania) were roasted at 130 °C for 45 min and ground in a Mully Top stone mill (ICB Tecnologie, Calcinato, Italy) for 2 h.

### 2.2. Date Paste

The date paste was obtained by soaking pitted dates in water (ratio 1:1) for 24 h at 4 °C. The hydrated dates together with the soaking water were ground in a Beko HBG5100W (Arçelik, Istanbul, Turkey) food processor for 5 min at normal speed. The resulting paste was filtered through a fine sieve to obtain a smooth, homogeneous paste.

### 2.3. Oleosomes Extraction

The raw sunflower seed kernels were soaked in water using a ratio of 1:1 (kernel: soaking water) for 24 h at 4 °C. The soaking water was removed and replaced by fresh water in a 1:1 ratio, then ground in a food processor and subjected to filtration through 4 layers of cheesecloth. The filtered liquid was centrifuged in a Hettich Universal 320R (Andreas Hettich, Tuttlingen, Germany) centrifuge at 9000 rpm for 1 h. The lipid phase was then collected and further used as oleosomes.

### 2.4. Ice Cream Mixes (ICM) and Ice Creams (IC) Preparation

Date paste, sunflower tahini, pumpkin seed tahini, refined sunflower oil (Ardealul S.A., Satu Mare, Romania), and oleosomes were used as materials to obtain the product. Emulsions were obtained using the IKA Ultra-Turrax (IKA-Werke, Staufen im Breisgau, Germany) homogenizer by mixing tahini, water, oil, and oleosomes. Homogenization was performed for 3 min at a speed of 15,000 rpm, obtaining three types of emulsions with varying proportions of oleosomes. According to the manufacturer’s specifications, homogenization using an IKA Ultra-Turrax (IKA-Werke, Staufen im Breisgau, Germany) for 2–5 min. typically produces emulsion fineness in the range of approximately 1–10 µm.

The ice cream mix (ICM) was formulated with 33% date paste and 67% emulsion, and the final composition of the ICM is shown in Table 1. These two components were homogenized for 2 min at a speed of 15,000 rpm and aged for 24 h at 4 °C, then frozen in HEINNER GLACE HICM-150WHYG (Network One Distribution, Bucharest, Romania) for 60 min at −20 °C. After freezing, the IC was stored at −19 °C.

### 2.5. Ice Cream Analysis

#### 2.5.1. Overrun Analysis

The overrun was determined using a 65 mL plastic container, comparing the mass of the ICM and the IC, in the same fixed volume.

The overrun was calculated using the following equation:

- Overrun [%] = $\frac{M1-M2}{M1}$ × 100
- M1—mass of the ICM.
- M2—mass of the IC.

#### 2.5.2. Flow Behavior of Ice Cream Mixes

Viscosity was determined at 5 °C using the Anton PAAR MCR 302 rheometer (Anton Paar, Austria), varying the shear rate (0.01–100 1/s). This analysis allows a detailed viscosity profile to be outlined, providing essential information about the flow properties of the ICM. This directly influences technological stages such as homogenization and freezing, as well as the consistency and texture of the finished product.

The rheological behavior data of the ICM from the ascending segment were fitted to the Ostwald–de Waele model:

τ = K(ɣ)^n^, where τ = shear stress (Pa s), ɣ = shear rate (s^−1^), K = consistency index (Pa s^n^), and n = flow behavior index [18].

#### 2.5.3. Textural Properties of Ice Cream Samples

The Brookfield CT3 Texture Analyzer (AMETEK Brookfield, Middleborough, MA, USA) was used to determine the hardness, adhesiveness, and cohesiveness of the IC samples. The samples were placed in containers with a diameter of 48 mm and a height of 22 mm, then stored at −20 °C. The texture profile analysis (TPA) test was conducted with the TA41 probe, with a load of 0.05 N, a speed of 1 mm/s, and a set depth of 10 mm. The analysis was performed at least in duplicate at three different temperatures, namely −16 °C, −12 °C, and −9 °C.

#### 2.5.4. Colorimetry Analysis

The color parameters were determined using the NR0 portable colorimeter (3NH, Shenzhen, China). The instrumentation was first calibrated using a standardized white reference plate to establish a consistent baseline for the L*, a*, and b* coordinates. The CIELab color system was used to evaluate the color of the product, which allows for accurate characterization of the chromatic parameters. Thus, the brightness of the samples is expressed by the L* parameter, with values ranging from 0 (absolute black) to 100 (perfect white), indicating how light or dark a sample is. The a* parameter reflects color variations on the green–red axis, with negative values for shades of green (a^−^) and positive values for shades of red (a^+^). Similarly, the b* parameter expresses variations on the yellow–blue axis, with negative values indicating shades of blue (b^−^) and positive values corresponding to shades of yellow (b^+^) [19].

The chroma (C*) was calculated with the following mathematical function:

$$C^{*}=\sqrt{\left(a^{2}+b^{2}\right)}.$$

The hue angle (H*) was calculated with the following mathematical function:

$$H^{*}={tg}^{-1}(\frac{a^{*}}{b^{*}})\;(\mathrm{expressed in degrees}).$$

Overall colorimetric difference between two samples (ΔE) was calculated with the following mathematical function:

$${\Delta E}^{*}=\sqrt{{{(\Delta L}^{*})}^{2}+{{(\Delta a}^{*})}^{2}+{{(\Delta b}^{*})}^{2}}$$

where ΔL is the difference in lightness (L*) between the two samples, Δa is the difference in the red–green coordinate between the samples, and Δb is the difference in the yellow–blue coordinate between the samples.

#### 2.5.5. Stability Assessment

##### Melting Time

A 10 g sample at a temperature of −18 °C was transferred to a watch glass and kept at room temperature (25 ± 1 °C). The sample was continuously monitored visually, and the time was measured starting at the moment of exposure to room temperature until the entire mass had completely melted.

##### Phase Separation

The samples to be analyzed were melted, then 7 mL of each was transferred to a sealed tube. The tubes were stored vertically at 4 °C for 7 days. At the end of the period, the presence or absence of phase separation was observed as an indicator of emulsion stability.

#### 2.5.6. Acceptability

The hedonic test (or acceptability test) is an effective method used in sensory analysis to evaluate consumer preferences for food products. The purpose of this test was to determine the degree of pleasure or acceptability of the IC, as influenced by the tahini type, by indicating an option on a standardized hedonic scale [20].

Evaluators, students of the Faculty of Food Science and Technology, Cluj-Napoca, were selected based on availability criteria. Two samples were used for testing, uniquely and anonymously labeled to ensure the objectivity of the evaluation: sample 634 (pumpkin tahini IC) and sample 249 (sunflower tahini IC), both formulated according to recipe ICM 0% in Table 1. Each sample was presented individually to the evaluators in clean bowls in a quantity of approximately 15 g. The order of serving was random to limit the impact of the sample position on the hedonic score. The panelists were asked to complete the evaluation form, shown in Table 2, indicating the overall acceptability of each attribute of the sample (aspect, smell, texture, taste, aroma, and overall appreciation) based on the hedonic scale.

#### 2.5.7. Statistical Analysis

The data for all analyses were statistically processed by calculating the arithmetic mean and standard deviation. All determinations were performed at least in duplicate to ensure the accuracy and reproducibility of the results. The results were expressed as mean ± standard deviation. All graphical representations of the data were created using OriginPro 2024 software.

Statistical significance was evaluated using a one-way analysis of variance (ANOVA). To evaluate the textural properties, a two-way analysis of variance (ANOVA) was conducted, utilizing temperature and oleosome concentration as the primary independent factors. To identify specific differences between sample groups, Tukey’s Honestly Significant Difference (HSD) post hoc test was applied for multiple comparisons. All statistical procedures were performed using Minitab 19, and differences were considered statistically significant at *p* < 0.05. Statistical significance was evaluated using a one-way analysis of variance (ANOVA).

## 3. Results

### 3.1. Degree of Aeration (Overrun)

The degree of aeration is one of the most important parameters in IC production, influencing the texture, consistency, and overall quality of the product [21]. The analysis of the degree of aeration (overrun) showed similar values for all three samples tested, namely (11.57 ± 0.12) for IC 0%; however, there were slightly higher values for the compositions containing oleosomes, namely (11.87 ± 0.21)% for IC 12% and (11.65 ± 0.19)% for IC24%. These values suggest a dense structure with low incorporated air content. Increasing oleosome content or improving the formulations may lead to higher values. An IC containing milk fat was reported to present an overrun of (18.37 ± 0.99)%, while the inclusion of 40% soybean oleosomes increased the parameter to (29.74 ± 0.47)%, suggesting that the purity of the oil bodies or the type of the proteins present in the structure might influence the ability to incorporate air in the product and the stabilization of the air bubbles [22]. Low overrun may result in a denser texture that may be perceived negatively by consumers and could reduce profitability at the industrial scale, since it leads to higher ingredient cost per unit volume and longer hardening times [3]. Low incorporated air could also lead to an intensely cold, sharp mouthfeel during consumption, as air can no longer serve as a thermal insulator [23].

### 3.2. Color Parameters

Color plays an essential role in consumers’ perception and acceptance of food products [24]. Color can influence the perception of a product’s attractiveness by forming expectations about taste and quality, which can have a significant impact on food choice and consumption [25].

IC color parameters are presented in Table 3. The L* values ranged from 52.86 ± 0.14 to 55.79 ± 0.18, indicating a relatively light color, with IC 0% being the brightest. The air bubbles in the product can scatter the light, meaning the inclusion of a larger quantity of air during the processing of the IC conducted to lower L* values. However, higher L* values are associated with greater visual appeal [24].

The a* values, which reflect the red–green axis, showed a gradual decrease from 7.53 ± 0.13 in I0% to 6.92 ± 0.14 in I24%, indicating a fading of reddish hues. These tones can be associated with ingredients such as date paste. Similarly, a decrease in a* values was also reported in a study which explored the replacement of milk fat with 50% oleosomes [22].

The b* values (yellow–blue axis) were relatively high in all three samples, ranging from 23.74 ± 0.09 in IC0% to 22.28 ± 0.15 in IC24%, indicating a consistent yellow hue. Yellow tones were most frequently associated with sour taste [24], but is also noted that orange (a combination of red and yellow) can be perceived as sweet, indicating that yellow hues can influence taste expectations and perceptions.

Chroma (C*) values showed a moderate color saturation typical of plant-based emulsions, ranging from 24.38 ± 0.09 to 23.77 ± 0.12. Oleosome-containing samples had a small drop in C*, indicating that the addition of oil bodies enhanced perceived whiteness and decreased color intensity.

The hue angle (H*) values, which range from 17.57 ± 0.32 to 15.90 ± 0.41, represent reddish-yellow tones that are in line with the pigments found naturally in date paste and sunflower seeds. A slight shift toward a more reddish tone is shown by a slight shift toward lower H* values in the samples with a higher oleosome content.

The overall colorimetric difference between IC0% and IC24% was 2.69. According to Wang et al. (2022), a difference in ΔE less than 3.7 is not perceivable by the human eye [22].

### 3.3. Texture

**Figure 1:**
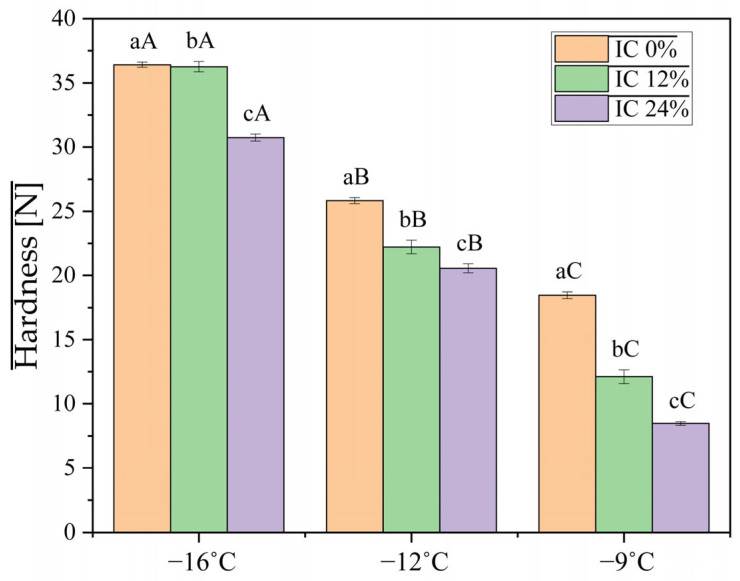
Hardness of ice cream mixes with different percentages of oleosomes as a function of temperature. Different lowercase letters indicate significant differences between concentrations at the same temperature, while different uppercase letters indicate significant differences between temperatures at the same concentration.

Hardness, shown in Figure 1, reflects the resistance of the IC to deformation and is influenced by fat content, internal network stability, and degree of aeration. Previous studies have shown that as the degree of aeration increases, hardness decreases [26]. The results obtained from the texture profile analysis (TPA) highlight significant differences between the three formulations. Sample IC 24%, formulated with a high content of oleosomes and, implicitly, vegetable proteins, showed hardness values between 8.48 ± 0.14 N at −9 °C and 30.74 ± 0.28 N at −16 °C, while sample IC 0% recorded hardness values between 18.48 ± 0.26 N at −9 °C and 36.43 ± 0.20 N at −16 °C. These results are consistent with Wang, Wang [22]’s findings, where hardness decreased as the percentage of oleosomes increased. In the IC matrix, temperature is the primary determinant of hardness (F = 2818.83), as it directly controls the ice phase volume and the rigidity of the fat network. The size and distribution of these ice crystals may be influenced by oleosome concentration, according to the very significant interaction (*p* = 0.000).

**Figure 2:**
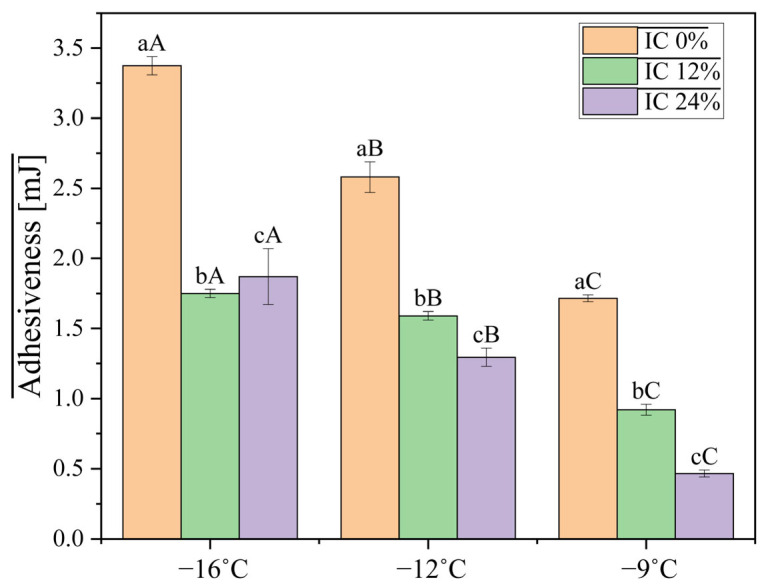
Adhesiveness of ice cream mixes with different percentages of oleosomes as a function of temperature. Different lowercase letters indicate significant differences between concentrations at the same temperature, while different uppercase letters indicate significant differences between temperatures at the same concentration.

Adhesiveness, shown in Figure 2, is measured as the force required to separate the IC from the surface of the probe. At −16 °C, IC 0% had an adhesiveness of 3.38 ± 0.06 mJ, which is higher than what was registered for IC 12% (1.75 ± 0.03 mJ) and IC 24% (1.87 ± 0.20 mJ). The values also decreased with the decrease in temperature. The inclusion of oleosomes decreased adhesiveness, differing from reports indicating that soybean oleosomes in IC formulations increase adhesiveness [25]. This can be explained by the different botanical sources of the oleosomes, differences in formulations, or the possibility that interactions between oleosomes and the other ingredients in IC affect adhesiveness differently. The concentration of oleosomes likely increases fat-mediated stickiness at different temperatures and reduces adhesiveness at lower temperatures by stabilizing fat droplets, according to the significant interaction (*p* = 0.008).

Lower adhesiveness values are desirable because they indicate a less sticky texture that is easier to handle and more pleasant from a sensory point of view. Studies show that increased viscosity of the mix contributes to higher adhesiveness values in some cases [26].

**Figure 3:**
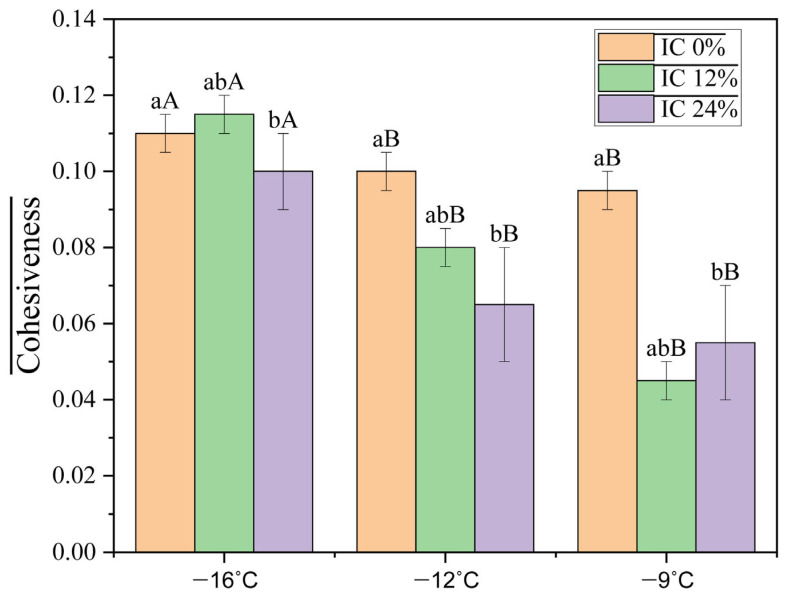
Cohesiveness of ice cream mixes with different percentages of oleosomes as a function of temperature. Different lowercase letters indicate significant differences between concentrations at the same temperature, while different uppercase letters indicate significant differences between temperatures at the same concentration.

Cohesiveness, shown in Figure 3, expresses the ability of IC to maintain its structural integrity during consumption. High values suggest a well-connected internal network formed by the interaction between proteins and fats. In the case of IC 24%, the cohesiveness of 0.10 ± 0.01 reflects a higher degree of stabilization, similar to the results obtained in another study for IC alternatives [27]. Ref. [28] also mentions that a viscous structure, formed by the binding of carbohydrates with water, contributes to increased cohesiveness and internal adhesion, which supports the better textural performance of IC 24%.

The oleosome–protein network creates a structural framework with the fat globules, as indicated by the interaction effect (*p* = 0.014) being such a powerful driver. At certain concentrations, the strength of this network varies with temperature, and the oleosomes create a more cohesive structure between ice crystals and air cells.

Gummines, a secondary textural factor derived from hardness and cohesiveness, were not influenced by the interaction of the factors (oleosome content %) and temperature of analysis, while intergroup differences occurred between ICO and IC24, with the gummines being decreased from 3.35 ± 0.15 N to 2.07 ± 0.27 N at −16 °C.

The value of these parameters gradually decreases as the product approaches melting, and the structure becomes softer, less cohesive, and less adhesive. This behavior is typical for IC and confirms that textural stability is influenced by both formulation and serving temperature.

### 3.4. Caloric Content

The energy value of the developed products, according to the data presented in Table 4, decreases as the oleosomes percentages increase.

The nutritional composition of IC 24%, presented in Table 4, shows a content of 18.87 g of fat and a slightly increased protein content of 5.80 g of protein, with an energy value of 245 kcal per 100 g of product, while IC 0% has a higher fat content and energy value of 32.47 g and 360 kcal, respectively, with a lower protein content of 3.90 g per 100 g product. IC 12% ranks between the other two samples with 302 kcal, 25.6 g fat, and 4.84 g protein.

Despite their high fat content, between 18.87 g and 32.47 g for IC 24% and IC 0%, respectively, the presented formulations offer a better nutritional profile than commercial sunflower-based options, as they have low saturated fat levels (1.28–3.76 g), unlike other sunflower alternatives (10 g) or the overall market average of 8 g, which is frequently elevated due to the predominant use of coconut oil. The proposed formulations are much more balanced than other commercially available options, placing them at the lower end of the 90% group of products on the market with sugar content over 10 g. The most important competitive advantage is the protein content (up to 5.8 g), which significantly exceeds the average of 2 g found in sunflower-based products and most of the databases analyzed [29].

### 3.5. Stability Assessment

#### 3.5.1. Melting Duration

**Figure 4:**
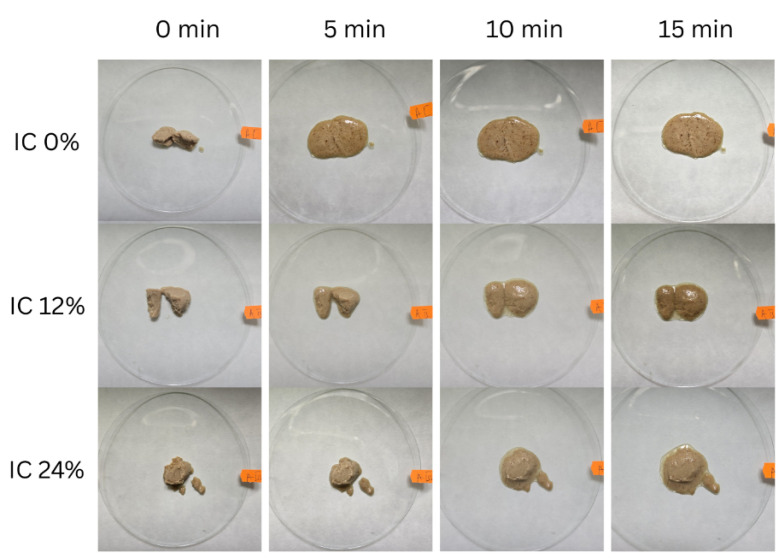
Visual evolution of the melting process.

Determining the melting duration revealed a clear correlation between the oleosome content and the thermal stability of the product. Figure 4 shows the dynamics of the melting process, where sample IC 0%, which does not contain oleosomes, had the shortest melting time (5 min), indicating a poorly stabilized structure susceptible to rapid melting at ambient temperatures.

In contrast, sample IC 24%, with the highest oleosome content, had the longest melting time (15 min), suggesting a significant stabilizing effect of these lipid–protein structures. Sample IC 12%, with an intermediate oleosome concentration, melted in 10 min, confirming this trend.

The formation of a fat network resulting from partial coalescence explains the melting delay process. This occurs when fat globules partially merge under shear force during freezing, forming large aggregates by migrating to the surface of the air bubbles. This network stabilizes the aggregates and forms a structural barrier that prevents serum leakage, even after the ice has melted. This increases the strength of the interfacial film. In addition, although the aggregate network is essential for maintaining shape in high-fat products, ice crystals provide stability by forming a complex pathway that prevents liquid drainage [30].

#### 3.5.2. Phase Separation

After 7 days of storing the samples in refrigerated conditions, no changes or destabilization were observed. From the absence of phase separation, we can deduce that proteins from the tahini, along with the oleosomes, formed emulsions that exhibit excellent stability during short-term storage, with the lipid phase remaining uniformly dispersed and retained in the product matrix, without migrating to the surface or separating from the aqueous phase. This suggests that the interfacial interactions formed by the oleosome membrane are strong enough to prevent emulsion destabilization under refrigeration conditions.

### 3.6. Viscosity

**Figure 5:**
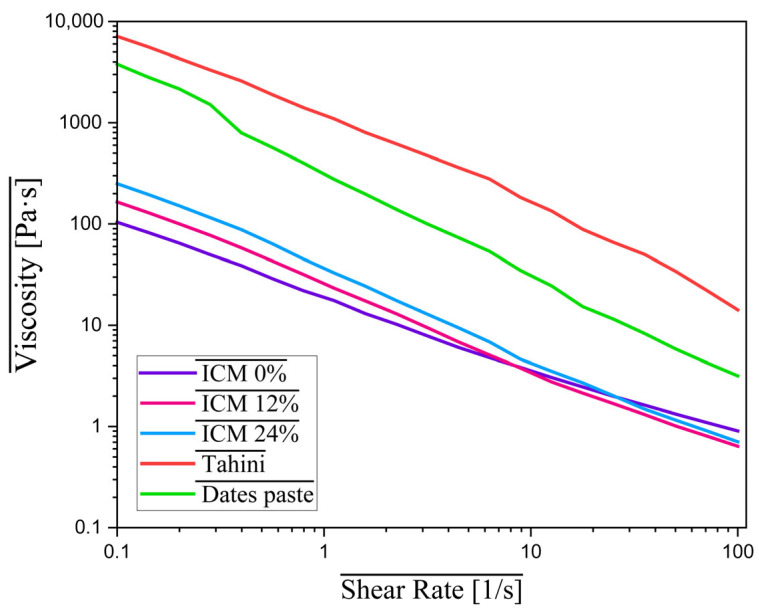
Flow behavior of ingredients (tahini, date paste) and ice cream mixtures.

The analysis of the rheological behavior of the raw materials used, oilseed paste and date paste, showed high viscosity across the entire shear rate range. All samples reacted similarly to the shear rate increase, namely, they exhibited shear thinning behavior, which has also been reported in other studies [31]. The values and evolution of viscosity for the raw materials are shown graphically in Figure 5, indicating that the date paste had an initial viscosity of 3796.95 Pa·s, decreasing progressively to 3.165 Pa·s as the shear rate increased. Similarly, the tahini had an even higher viscosity at the start of the test, 7119.3 Pa·s, which decreased to 14.20 Pa·s. This behavior reflects an increased concentration of solids and the presence of macromolecular components such as proteins and fibers.

The viscosity of ICM plays an essential role in determining texture, melting resistance, and the stability of air bubbles formed during the aeration process [26].

The viscosity of the formulated mixtures showed slightly different values, depending on the oleosome content. Sample ICM 0% had the lowest viscosity, starting at 104.09 Pa·s and decreasing to 0.90 Pa·s. ICM 12% recorded higher values, between 165.15 Pa·s and 0.64 mPa·s, while ICM 24% had the highest initial viscosity, 250.38 Pa·s, which decreased to 0.70 Pa·s.

This pseudoplastic behavior, characterized by a decrease in viscosity with increasing shear rate, is typical of ICM and reflects a well-organized internal structure. This property is beneficial in the technological process: it allows for easy flow during homogenization and freezing, but also contributes to maintaining a creamy, uniform, and stable texture in the final product [5,6].

The addition of sunflower oil bodies results in a microstructure where the oleosomes restrict the development of a continuous ice crystal matrix. By disrupting these ice-to-ice connections and limiting the movement of the unfrozen serum, the oleosomes prevent the development of a brittle, hard texture. Compared to IC 0%, the IC 24% had significantly lower hardness. The oil body samples had a higher initial mix viscosity, but the hardness of the frozen product was reduced. This shows that rather than the mix viscosity, the final texture is mostly determined by the interfacial stability of the oil bodies and their capacity to prevent the formation of big ice crystals.

According to ref. [31], IC with high viscosity has a greater resistance to melting, which correlates with ICM 24% having the highest viscosity of the three samples, while also having a higher resistance to melting. Elevated viscosity is known to restrict air incorporation during freezing, which may explain the limited overrun observed [6,32].

The K, n, and R^2^ values at different oleosome concentrations are presented in Table 5. Sample ICM 24% presented the highest K (35.812 ± 7.893), compared to the other two samples. At the same time, the same sample exhibited the highest viscosity, with values from 250.38 Pa·s, decreasing to 0.70 Pa·s. This behavior could be attributed to the higher oleosome concentration, which created a durable structural framework, yielding higher consistency index values.

Flow behavior index values (*n*) were less than 1 for all the samples, which confirms the shear thinning and non-Newtonian behavior that are characteristic properties of IC, especially for ICM and partially melted IC [32]. The model presents high R values for ICM 0%.

#### Hedonic Sensory Analysis

**Figure 6:**
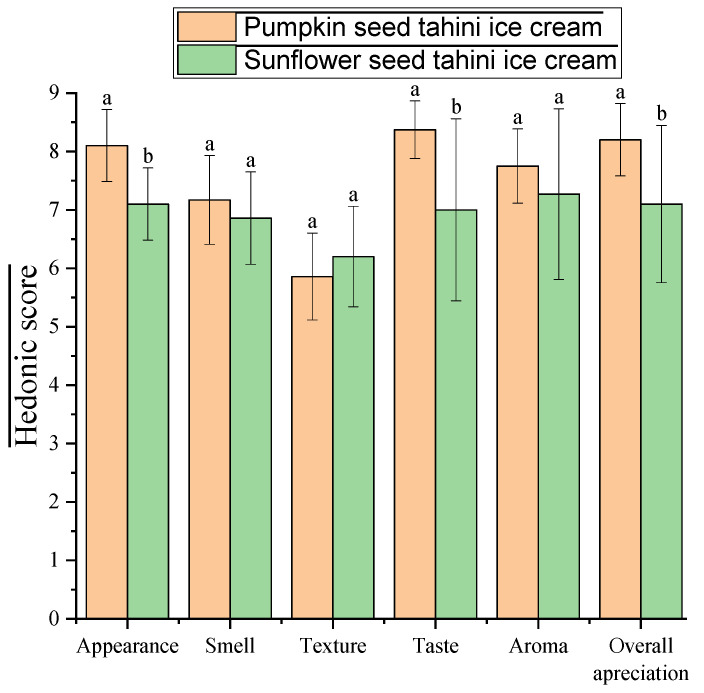
Hedonic test results for the sensory assessment of the pumpkin seed and sunflower seed tahini ice creams (identical letters indicate no significant difference (*p* > 0.05) among the samples).

Following the hedonic test on the two samples of IC, both formulated according to recipe ICM 0% from Table 1, there were notable differences in the panelists’ perception of the sensory attributes evaluated, due to the different tahini type. The pumpkin seed tahini IC consistently obtained higher average scores, as shown in Figure 6, for most attributes: appearance: 8.10 ± 0.62, taste: 8.38 ± 0.49, aroma: 7.76 ± 0.64, and overall appreciation: 8.21 ± 0.62. In contrast, the sunflower tahini sample obtained lower scores for these attributes, especially for taste, 7.00 ± 1.56, and overall appreciation, 7.10 ± 1.35. This contrast suggests a clear preference of the evaluators for the sensory profile offered by pumpkin tahini.

Both samples received relatively low texture scores, 5.86 ± 0.74 for pumpkin tahini and 6.21 ± 0.86 for sunflower tahini, which indicates the need to improve this characteristic; therefore, the addition of oleosomes could address this problem. It is possible that the current texture does not meet consumer expectations regarding the creaminess of IC.

The most significant differences between the two recipes were observed in terms of taste, aroma, and overall appreciation. For the sunflower tahini sample, the standard deviations for taste: ±1.56, aroma: ±1.46, and overall appreciation: ±1.35 are significantly higher than for pumpkin tahini, where these values are much lower: ±0.49, ±0.64, and ±0.62, respectively. This suggests a polarized consumer response: some panelists rated the sunflower tahini sample highly, while others rated it lower, most likely due to a more intense or atypical flavor profile for such a dessert. In contrast, for the pumpkin tahini sample, these three attributes showed clear consistency, suggesting a more homogeneous perception and more stable overall acceptability, but also that the overall appreciation was directly influenced by its aromatic profile. This high variability signals a polarizing sensory profile, which could be perceived as too intense or unusual for a frozen dessert.

## 4. Conclusions

The use of oleosomes extracted from oilseeds as structural agents in sugar-free, vegan frozen desserts to substitute fat and artificial emulsifiers and to obtain a nutritionally enhanced plant-based product showed potential but also some drawbacks that require more research.

In terms of energy value, fat intake, proteins, and fiber, the product with the highest oleosome concentration was above market average. Higher oleosome concentration decreased fat intake; by raising the concentration even further, the product might be more balanced and have lower fat percentages than the market average. With 10.81 g of sugar, it has placed at the lower end of the over 90% group of products on the market with over 10 g of sugar. The fats and proteins, derived mainly from tahini and oleosomes, provided both nutritional enrichment and functional enhancement of the IC structure, thereby improving its overall value.

With temperature serving as the main factor, a higher concentration of oleosomes resulted in a decrease in hardness. The concentration of oleosomes also decreases adhesiveness at lower temperatures by stabilizing fat droplets. The oleosomes produce a more cohesive structure between ice crystals and air cells. The oleosome–protein network creates a structural framework with the fat globules; at certain concentrations, the strength of this network varies with temperature.

Instrumental analyses complemented the sensory evaluation, highlighting a light color with reddish and yellow hues. The melting time was found to be directly influenced by the presence of oleosomes, as the product is stabilized and serum leakage is avoided by the formation of a fat network during freezing, with samples with a higher content showing increased melting stability. The evaluation of the physical stability of the emulsions showed that no phase separation occurred after 7 days of refrigeration, indicating that the oleosome membrane’s interfacial interaction is strong enough to prevent emulsion destabilization.

The low overrun, which could result in a denser product with a sharp cooling sensation and be linked to higher prices per unit, is one of the limitations. Acceptability analysis showed that the texture is another drawback that may be fixed by increasing air incorporation, which would result in a less dense product with better textural properties.

An interesting approach could be replacing date paste with local fruits that are rich in carbohydrates, which would maintain the role of a natural sweetener while also supporting the use of local ingredients. Raisins, plums, or even dehydrated apples could be viable alternatives to date paste, with benefits in terms of both supply chain sustainability and product adaptation to local specificities. These ingredients could also contribute to shaping a regional, authentic character that would differentiate the product in a market increasingly interested in the local origin of food.

## Figures and Tables

**Table 1:** Composition of the ice cream formulations.

	ICM 0%	ICM 12%	ICM 24%
Date paste	33	33	33
Tahini	15.45	15.45	15.45
Sunflower oil	24.74	12.36	-
Water	26.81	26.81	26.81
Oleosomes	-	12.36	24.74

**Table 2:** Evaluation form.

Question	Possible Answer
How much do you like or dislike the aspect of the sample?	1—extremely unpleasant, 2—very unpleasant, 3—unpleasant, 4—slightly unpleasant, 5—indifferent, 6—slightly pleasant, 7—pleasant, 8—very pleasant, and 9—extremely pleasant.
How much do you like or dislike the smell of the sample?	
How much do you like or dislike the texture of the sample?	
How much do you like or dislike the taste of the sample?	
How much do you like or dislike the aroma of the sample?	
Overall, how much do you like or dislike the sample?	

**Table 3:** Color parameters and overrun of plant-based ice cream mixtures.

Sample	L*	a*	b*	C*	H*
IC 0%	55.79 ± 0.18 ^a^	7.53 ± 0.13 ^a^	23.74 ± 0.09 ^a^	24.38 ± 0.09 ^a^	17.57 ± 0.32 ^a^
IC 12%	53.72 ± 0.12 ^b^	7.25 ± 0.1 ^a^	23.14 ± 0.07 ^a^	24.30 ± 0.04 ^a^	16.52 ± 0.27 ^b^
IC 24%	52.86 ± 0.14 ^c^	6.92 ± 0.14 ^b^	22.28 ± 0.15 ^b^	23.77 ± 0.12 ^b^	15.90 ± 0.41 ^b^

Data are expressed as mean ± SD. Different letters (a, b, and c) in the same column reveal significant differences (*p* < 0.05) among the samples. L* brightness of the samples, a*—color variations on the green–red axis, b*—color variations on the yellow–blue axis, C*—Chromaticity, H*—Hue.

**Table 4:** Caloric content estimation based on the ingredients’ nutritional value [per 100 g product].

Sample	Energy Value	Fat	Of Which Saturated Fatty Acids	Carbohydrates	Of Which Sugars	Protein	Fiber
	[kcal]	[g]	[g]	[g]	[g]	[g]	[g]
IC 0%	360	32.47	3.76	12.95	10.81	3.90	1.60
IC 12%	302	25.60	2.52	12.95	10.81	4.84	1.60
IC 24%	245	18.87	1.28	12.95	10.81	5.80	1.60

**Table 5:** The Ostwald de Waele parameters of the ice cream mixtures.

Sample	K [Pa s^n^]	n	R^2^
ICM 0%	19.325 ± 3.650 ^b^	0.298 ± 0.014 ^a^	0.978 ± 0.003
ICM 12%	25.389 ± 6.780 ^ab^	0.169 ± 0.026 ^b^	0.953 ± 0.020
ICM 24%	35.812 ± 7.893 ^a^	0.119 ± 0.025 ^b^	0.924 ± 0.023

Data are expressed as mean ± SD. Different letters (a, b) in the same column reveal significant differences (*p* < 0.05) among the samples.

## Data Availability

The original contributions presented in this study are included in the article. Further inquiries can be directed to the corresponding author.
